# Impact of maternal and neonatal factors on mortality in the first six weeks of life: A comprehensive cohort study in Kinshasa, DR Congo

**DOI:** 10.1371/journal.pgph.0005989

**Published:** 2026-08-21

**Authors:** Francis K. Kabasubabo, Holy Bezanahary, Julien Magne, Pierre Z. Akilimali

**Affiliations:** 1 Inserm U1094, IRD UMR270, CHU Limoges, EpiMaCT-Epidemiology of Chronic Diseases in Tropical Zone, Institute of Epidemiology and Tropical Neurology, Omega Health, University of Limoges, Limoges, France; 2 National Public Health Institute, Kinshasa, Democratic Republic of Congo; 3 Patrick Kayembe Research Center, Kinshasa School of Public Health, University of Kinshasa, Kinshasa, Democratic Republic of Congo; 4 Department of Internal Medicine, Limoges University Hospital, Limoges, France; 5 Department of Nutrition, Kinshasa School of Public Health, University of Kinshasa, Kinshasa, Democratic Republic of Congo; PLOS: Public Library of Science, UNITED STATES OF AMERICA

## Abstract

The Democratic Republic of Congo (DRC) is among the sub-Saharan African countries with a high neonatal mortality rate. Various interventions have been implemented to reduce this mortality rate, including the establishment of free maternity policy. This study evaluated the neonatal and maternal factors influencing infant mortality at 6 weeks of life. A prospective cohort study was conducted on infants recruited between March and July 2024 from maternity wards in Kinshasa, DRC. The cohort was followed for up to 6 weeks with home visits. The statistical analysis used Kaplan-Meier and adjusted Cox models. The multivariate Cox model was fitted with variables selected by LASSO and missing data were imputed using multiple imputation according to Rubin’s rules. Among a cohort of 2,383 infants followed, 3.2% had a low-5 minutes Apgar score and 96.8% had a normal Apgar score. One of five babies with low 5-minutes Apgar scores died by the age of six weeks. Overall, the early postnatal mortality was 2.2% (95% CI: 1.67-2.85) with an overall mortality incidence of 0.53 deaths per 1,000 person-days. Multivariate Cox regression analysis with multiple imputation identified statistically significant increases in the associations of early postnatal mortality risk for low-5 minutes Apgar score (aHR: 9.96; 95% CI: 7.01-14.17), low birth weight (aHR: 3.57; 95% CI: 2.39-4.72) and prematurity (aHR: 1.97% CI: 1.26-3.12). Conversely, antenatal visits were a protective factor, with each additional antenatal visit associated with a 19% reduction in the risk of infant mortality (aHR: 0.81; 95% CI: 0.66-0.99). These results underscore the crucial importance of high-quality obstetric and neonatal care, as well as antenatal monitoring, for improving newborn survival. They call for strengthened antenatal care, improved screening and management of at-risk newborns in healthcare facilities, and the integration of these indicators into strategies for reducing neonatal mortality in resource-limited settings.

## Introduction

Over the past twenty-five years, global data have shown a halving of infant mortality worldwide [1]. Although under-five mortality declined substantially worldwide, reductions in neonatal mortality have progressed more slowly. In 2023, about fifty percent of all deaths among children under five transpired during the neonatal period, highlighting its substantial impact on worldwide child mortality. Despite progress, stark disparities persist, as the vast majority of these deaths still occur in South-East Asia and sub-Saharan Africa (SSA) [1,2].

Neonatal mortality remains a considerable public health concern, particularly in low- and middle-income countries (LMICs). According to the World Health Organization (WHO), about 2.3 million newborns died during their first month of life in 2023, corresponding to a global neonatal mortality rate of 17 deaths per 1 000 live births (LB) [3]. In SSA, the neonatal mortality rate (NMR) reached 26 per 1 000 LB, while in the Democratic Republic of Congo (DRC) it was 19 per 1 000 LB [4]. These figures remain above the Sustainable Development Goal (SDG) 3.2, which aims to reduce the NMR to 12 deaths or fewer per 1 000 LB by 2030 [5].

In DRC, despite national efforts, neonatal mortality remains high due to multiple challenges related to both the health system and the socioeconomic context. Access to quality antenatal, intrapartum, and postnatal care remains unequal, particularly among vulnerable populations. Health care facilities (HCFs) frequently face shortages of qualified healthcare personnel, essential medicines, neonatal resuscitation equipment, and specialized services for newborn care. These difficulties are exacerbated by socioeconomic inequalities that delay seeking care and restrict access to fully insured antenatal, obstetric, and postnatal services [6–9].

To accelerate progress toward this target, the DRC launched a policy of free maternity care initiative in 2023, designed to remove barriers-especially financial to maternal and neonatal healthcare. The policy was launched in Kinshasa and subsequently extended to other provinces (including Kongo central, South Kivu and Kasai-Oriental). This policy provides free healthcare for pregnant women until delivery and for newborns up to one month of age, aiming to reduce preventable neonatal deaths.

The neonatal period is widely recognized as one of the most critical phases of human life [10]. Early evaluation of newborn health is therefore essential. Among available tools, the Apgar score, developed by Virginia Apgar in 1952, remains the most universally used method to assess a newborn’s clinical condition at one and five minutes after birth [11–14]. This score rapidly evaluates five physiological parameters - skin color, heart rate, respiratory effort, muscle tone, and reflex irritability. A low Apgar score at one minute often indicates immediate distress due to intrapartum events, whereas the score at five minutes is a stronger predictor of short- and medium-term outcomes, including neonatal survival, neurological development, and long-term morbidity [14–17].

In resource-limited settings, perinatal complications such as birth asphyxia, prematurity, and neonatal infections remain the leading causes of neonatal death [3,18]. These risks are exacerbated by structural challenges, including the shortage of skilled birth attendants, delayed access to postnatal care, and limited neonatal resuscitation capacity [19]. In such contexts, the Apgar score can serve not only as a bedside clinical tool but also as a strategic triage indicator for early intervention.

Although the five-minute Apgar score has been widely studied as a predictor of early neonatal mortality, data regarding its association with survival during the first six weeks of life remain limited, particularly in SSA [20–22]. Most published studies have focused on mortality within the first 7 or 28 days after birth, leaving a knowledge gap concerning survival patterns during the extended neonatal period. Furthermore, studies trying to understand the impact of maternal factors are still lacking in so far as some characteristics of the pregnancy could influence the newborn’s outcomes.

In the DRC, maternal and newborn health programs follow the “three-six approach” recommended by the National Reproductive Health Program (NRHP), which schedules postnatal visits at six hours, six days, and six weeks after birth. Consequently, the national reference period for neonatal mortality extends to 42 days (six weeks) rather than 28 days. This difference is critical, as many infant deaths occur after hospital discharge, often in contexts of limited follow-up and restricted access to emergency care.

This study aims to assess the mortality rate of newborns at six weeks and to identify independent predictors of this mortality, specifically the five-minute Apgar score, birth weight, and gestational age in Kinshasa, DRC. The results should inform evidence-based health interventions, improve neonatal care practices, and support the development of context-appropriate strategies aligned with global goals for reducing preventable infant mortality. We hypothesize that infants with a low five-minute Apgar score (< 7) have a higher risk of death during the first six weeks of life than those with a normal score (≥ 7), independent of potential confounding factors such as gestational age, birth weight, sex, and maternal characteristics.

## Methods

This prospective cohort study was conducted over a period extending from March 15, 2024, to July 31, 2024. Dyads mothers and infants were recruited at birth and followed up until the age of 6 weeks. The newborns included in the study were those who were born alive in the participating maternity units, whose Apgar score at 5 minutes was recorded in the partograph or medical record, and whose parent/guardian had given consent for follow-up. Newborns with a congenital malformation visible at birth or diagnosed immediately after delivery were not included in the initial cohort. This exclusion aimed to avoid confusion between neonatal complications related to a congenital anomaly and those attributed to the obstetric, maternal, or neonatal factors.

This study was conducted in Kinshasa, the capital of the DRC, a rapidly growing megacity whose healthcare system is organized into health zones encompassing public, private, and confessional facilities. According to the Ministry of Health’s 2022 annual statistics, published in 2023, Kinshasa’s healthcare system comprises 35 health zones, including 413 health areas, and has 1,021 primary care facilities, one secondary general referral hospital, and five tertiary general referral hospitals (national and university clinics). Despite the density of healthcare services, their distribution remains uneven, and the quality of services varies across the different districts.

This study included a total of 346 HCFs in Kinshasa and focused on facilities providing free maternity care as well as other HCFs not covered by this policy. All facilities providing free maternity care (251) were exhaustively included in the study. For facilities not providing free maternity care, a list of available HCFs was used. From this list, HCFs were selected using a simple random sampling procedure. These facilities were of the same standard and as comparable as possible to those offering free maternity care, particularly in terms of location (urban or rural), the level of obstetric care available (presence of a delivery room, availability of cesarean sections, basic neonatal care), and the annual volume of deliveries.

### Variables

The primary outcome was neonatal survival to six weeks. Time zero was defined as birth, and the date of death was accurately recorded from hospital records for death occurring during the mother’s postpartum period in the hospital. For death occurring after discharge from the maternity ward, the information was verified through telephone follow-up with families and verbal autopsy. Infants who had not died by the end of the follow-up period were censored at the date of the last documented visit or contact.

The covariates and potential confounding factors taken into account were neonatal characteristics such as Apgar score, birth weight, gestational age, and sex. The maternal variables taken into account were age, maternal education, parity, antenatal visits and the mode of delivery. In addition, the HCF was coded as 1 (“Free maternity) for those providing free maternity care and coded as 0 (“no free maternity”) for those not providing free care. HCFs have been groups into primary level (health center, reference health center) and secondary/tertiary level (general reference hospitals, university clinics). Birth weight was measured within one hour of the delivery using a mechanical neonatal scale with a slider and reported in grams. The birth weight was categorized as < 2,500g (coded 1 “low birth weight”) and ≥ 2,500g (coded 0 “normal birth weight”). Gestational age was determined by the availability of at least one ultrasound scan and was categorized as < 37 weeks of amenorrhea (WA) (coded 1 “prematurity”) and ≥ 37 WA (code 0 “born at term”). The Apgar score at 5 minutes has categorized as low Apgar (Apgar < 7) and normal Apgar (Apgar ≥ 7) [23,24]. The time variable was calculated from the date of enrolment to the end point (death or end of the study).

### Data collection, monitoring and evaluation of results

Fifty research investigators, all female healthcare professionals with a background as doctors or nurses, were recruited and trained to conduct data collection in the HCF, as well as to follow up with women and their newborns at home after their discharge from the maternity ward. The research team had the phone numbers of the maternity services of all the HCF included in the study. Every day, the research investigators responsible for a specific area made phone calls at 8:30 a.m. to check for the possible admission of new women into the HCF for childbirth. The phone call to the HCF regarding new admissions was for recruitment purposes in the study. Newborns were recruited from selected HCFs, and recruitment was systematic throughout the cohort inclusion period. Infants were followed prospectively from birth until 42 days of life. Follow-up was conducted through a combination of in-facility monitoring during the mother’s postpartum stay and post-discharge follow-up using scheduled phone calls and/or home visits. Information on vital status was collected at predefined time points during the follow-up period.

Deaths were ascertained through multiple sources, including hospital medical records for in-facility deaths and confirmation by household contact (telephone interviews or home visits) for deaths occurring after discharge. Where possible, additional verification was carried out using available health facility records or reports from community health workers.

Lost to follow-up (LTFU) was defined as inability to ascertain the vital status of the infant after repeated contact attempts. These cases were censored at the date of last known contact. Efforts were made to minimize LTFU through repeated phone calls and tracing procedures in cases of participant migration. Cases of participants LTFU were documented but not excluded from the main analyses, in order to minimize selection bias and ensure the cohort remained representative.

### Data analysis

A descriptive analysis was conducted to characterize the mothers and infants included in the cohort. For continuous variables, mean and standard deviation (SD) were calculated following their normal distribution; for categorical data, proportions and their respective 95% confidence intervals (CI) were calculated. We used the chi-square test and Fisher’s exact test when appropriate. The proportions were calculated, with death being the main outcome variable. The incidence rate of recorded deaths was determined per 1,000 patient-days (p-d) from the date of inclusion.

Survival was assessed using the Kaplan-Meier method, with curve comparisons using the log-rank to confirm differences in trends. The association between the explanatory variable and mortality was modeled using proportional Cox models, which were then adjusted. A regression approach penalized by the Least Absolute Shrinkage and Selection Operator (LASSO) was used. LASSO was applied to all candidate variables (5-minute Apgar score, birth weight, gestational age, infant’s sex, maternal age, maternal education level, parity, antenatal visits, socioeconomic status, and HCF group). The selection of the regularization parameter (lambda) was performed using 10-fold cross-validation. The variables retained by LASSO were then included in the multivariate Cox model with the clustering of facility.

The interactions between the antenatal visits and gestational age, Apgar score at 5 minutes and low birth weight were not significant and were not included in the model. The absence of detected interaction suggests that the reported associations are homogeneous across the sample, although we cannot exclude the possibility of interactions in other contexts or with large samples.

Compliance with the risk proportionality assumption was verified using the Schoenfeld residual proportionality test (refer to S1 Table). The proportional risk assessment showed compliance with this assumption, as illustrated in Fig A, B, C, D and E in S1 Appendix. Regarding the goodness of fit of the final model, it was observed that the risk function closely follows the bisector, except for very large values time. In summary, we would conclude that the final model fits the data adequately (refer Fig F in S1 Appendix). Multicollinearity was assessed using the variance inflation factor (VIF), which has a value of 1.33.

The missing data was imputed using the Markov Chain Multiple Imputation (MICE) method, assuming that the data was missing at random (MAR) based on the observed variables. The imputation model included all the variables from the analytical model (birth weight, gestational age, mother’s education level, antenatal visits). The analyzes were repeated on 10 imputed datasets, then aggregated according to Rubin’s principles [25]. A significance threshold of p < 0.05 was set to ensure the robustness of the results. The results were presented as hazard ratios (HR) with 95% CI. The number of missing data is indicated in S2 Table: out of 2,383 mother-child dyads, 364 data were missing regarding gestational age and 37 mothers lacked information on antenatal visit follow-up. All data were analyzed using Stata 18 SE (StataCorp, College Station, TX, USA), except for the Kaplan-Meier plots, which were generated using R software version 4.5.2. All tests were two-tailed with 95% confidence intervals and considered statistically significant when p-value < 0.05. Dataset can be found in open source framework: https://doi.org/10.17605/OSF.IO/MYTPA.

### Ethical considerations

As this study involved human participants, it was conducted according to the guideline of the Declaration of Helsinki. The necessary authorizations were obtained from the competent health authorities in the city province of Kinshasa, namely the head of the Kinshasa Health Division, the chief medical officer of the health zone, and the directors of the selected hospitals. An informed verbal consent was obtained from the mothers before their inclusion. Verbal consent was documented by the investigator on the data collection form by checking a specific box confirming that the study information had been provided, that the participant’s questions had been satisfactorily answered, and that the participant had voluntarily agreed to participate. No data was collected before obtaining consent. This verbal consent procedure, including its documentation method, was reviewed and approved by the Research Ethics Committee of the School of Public Health at the University of Kinshasa (ESP/CE/152/2023).

Data confidentiality and the possibility of withdrawing at any time were guaranteed. Participants under 18 years of age were considered as emancipated minors [26,27], and the requirement for consent from minors was waived by the ethic committee. To ensure confidentiality, we deidentified the variables in the database before the analyzes. The use of the results of this study will be strictly limited to the achievement of its objectives, and the authors declare that they have no conflicts of interest.

## Results

### Participants

**Fig 1:**
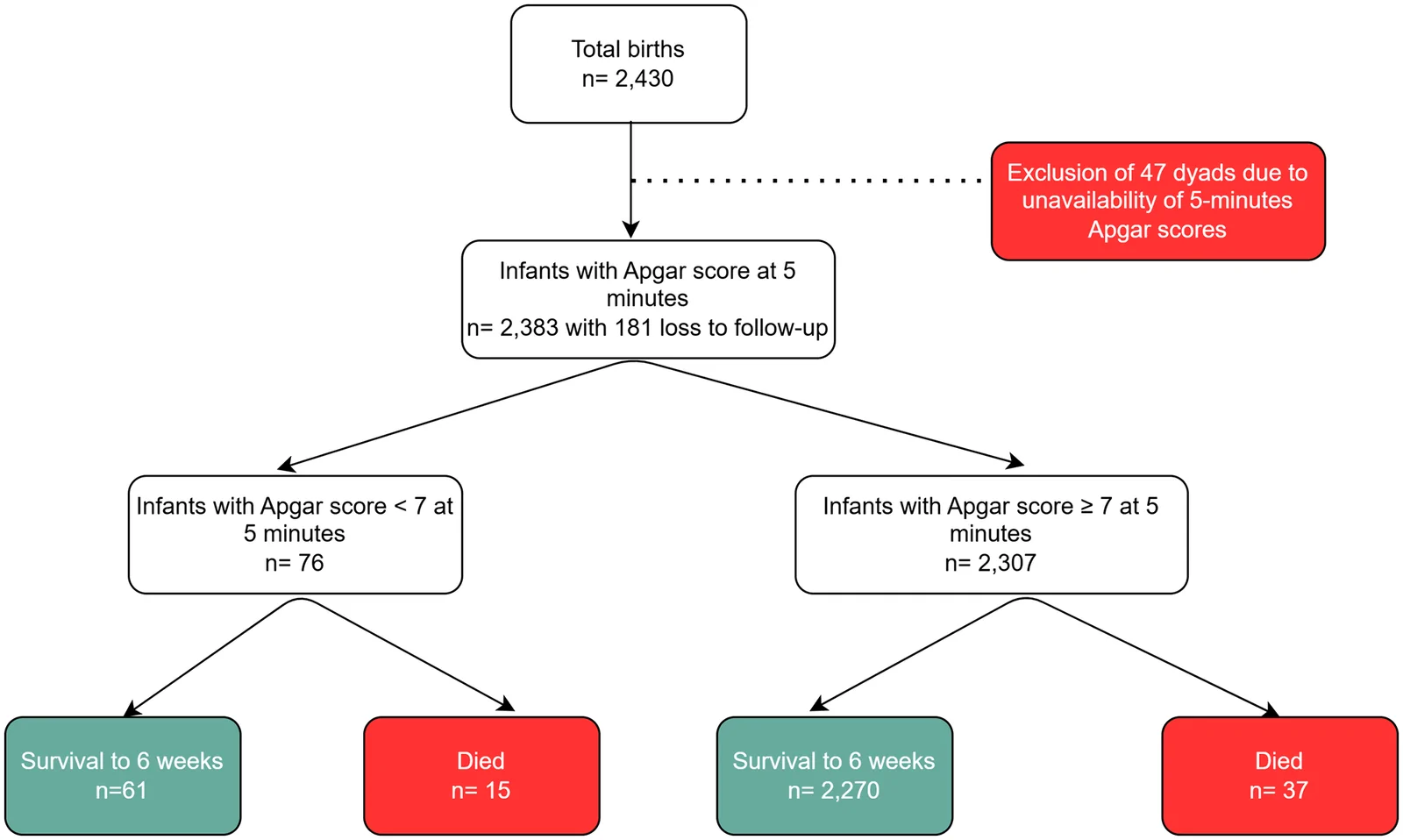
Flow diagram of participants.

Overall, 2,430 infants recruited at birth were followed, of which only 2,383 were eligible for inclusion in this present study (See Fig 1).

### Descriptive cohort characteristics

Among 2,430 infants followed, 76 infants had a low Apgar score (3.2%) and 2,307 had a normal Apgar score (96.8%).

The proportion of infants with low-5 minutes Apgar scores was higher among infant born with low birth weight (LBW) compared to those born with normal birth weight (7.8 vs. 2.8%; p = 0.01). For gestational age, there is no difference between infants born prematurely (gestational age < 37 weeks) and those born at term. Women who had no education or had a primary education level were more likely to give birth to a child with a low-5 minutes Apgar scores compared to those who had a secondary/university education (4.1 vs. 2.6%; p = 0.045). The proportion of infants with low-5 minutes Apgar scores was higher among infants born by cesarean section compared to those born vaginally (6.7 vs. 2.8%, p = 0.001). The other characteristics were statistically similar (Table 1).

**Table 1:** Descriptive characteristics of the study cohort, according to Apgar score at 5 minutes from March to July 2024, Kinshasa.

	Total	Apgar < 7		Apgar ≥ 7		p_value
		n	%	n	%	
**Healthcare facilities characteristics**						
Group of maternity						1,000
No free maternity	878	28	3.2	850	96.8	
Free maternity	1505	48	3.2	1457	96.8	
Management authority						0.657
Government	484	16	3.3	468	96.7	
Confessional	841	30	3.6	811	96.4	
Private/ NGO	1058	30	2.8	1028	97.2	
Type of HCF						0.339
Primary	1226	35	2.9	1191	97.1	
Secondary/ Tertiary	1157	41	3.5	1116	96.5	
**Maternal and household characteristics**						
Mother’s age (years) (mean ± SD)	27.71 ± 6.24	28.38 ± 6.92		27.69 ± 6.22		0.343
Mother’s age (years)						0.242
15-19	238	8	3.4	230	96.6	
20-34	1772	51	2.9	1721	97.1	
35-49	373	17	4.6	356	95.4	
Marital status						0.907
In union	2026	65	3.2	1961	96.8	
Alone	356	11	3.1	345	96.9	
Maternal education						0.045
None/ Primary	928	38	4.1	890	95.9	
Secondary/ University	1455	38	2.6	1417	97.4	
Household wealth index						0.092
Poorest/ Poor	84	1	1.2	83	98.8	
Middle	411	7	1.7	404	98.3	
Richer	815	23	2.8	792	97.2	
Richest	844	34	4.0	810	96.0	
**Obstetrical characteristics**						
Parity						0.814
Primiparous	788	27	3.4	761	96.6	
Pauciparous	1025	30	2.9	995	97.1	
Multiparous	570	19	3.3	551	96.7	
Mean of ANC ± SD	3.37 ± 0.85	3.39 ± 0.82		3.36 ± 0.85		0.109
ANC visits						0.922
Less than 4 ANC	1011	31	3.1	980	96.9	
4 ANC and more	1335	40	3.0	1295	97.0	
Gestational age (WA)						0.705
< 37	488	18	3.7	470	96.3	
37-42	1531	51	3.3	1480	96.7	
Mode of delivery						0.001
Vaginal	2130	59	2.8	2071	97.2	
Cesarean	253	17	6.7	236	93.3	
**Children’s characteristics**						
Sex						0.404
Boy	1234	43	3.5	1191	96.5	
Girl	1145	33	2.9	1112	97.1	
Mean of birthweight ± SD	3166.67 ± 541.53	3049.20 ± 686.13		3170.86 ± 535.85		0.054
Birth weight (gram)						0.001
< 2500	188	14	7.4	174	92.6	
≥ 2500	2195	62	2.8	2133	97.2	

SD = standard deviation.

WA = Week of Amenorrhea.

### Lost to follow-up and mortality

During the study period, the overall proportion of LTFU was 7.6% (95% CI: 6.60-8.73%) over six weeks of follow-up. Overall, the total number of person-days (p-d) followed was 99,137 with 9,290 p-d LFU.

Overall, the early postnatal mortality was 2.2% (95% CI: 1.67-2.85) with an overall incidence rate of 0.53 deaths per 1,000 p-d. (Table 2). One of five babies with low 5-minutes Apgar scores (n = 15) died by the age of six weeks (Table 2).

**Table 2:** Unajusted analysis.

Variables	n	Died		Person time	Incidence rate of death per	HR crude	95% IC	p_value
		Yes	No	(Days)	1,000 p-d			
Apgar score at 5 minutes								
< 7	76	15	61	2,842	5.28	13.60	7.46–24.78	<0.001
≥ 7	2,307	37	2,270	96,295	0.38	1		
Birth weight (gram)								
< 2500	188	17	171	7,570	2.25	5.89	3.30–10.51	<0.001
>=2500	2,195	35	2,160	91,567	0.38	1		
Gestational age (WA)								
< 37	488	20	468	20,084	1.00	2.27	1.28–4.03	0.005
≥ 37	1,531	28	1,503	63,898	0.44	1		
Infant sex								
Boy	1,234	26	1208	51,198	0.51	0.93	0.54–1.60	0.798
Girl	1,145	26	1,119	47,771	0.54	1		
Mother’s age (years)								
15 - 19	238	8	230	9,869	0.81	1		
20 - 34	1,772	37	1,735	73,720	0.50	0.62	0.29–1.33	0.218
35 - 49	373	7	366	15,548	0.45	0.56	0.20–1.53	0.256
Maternal education								
None/ Primary	928	35	893	38,358	0.91	3.26	1.83–5.82	<0.001
Secondary/ University	1,455	17	1,438	60,779	0.28	1		
Parity								
Primiparous	788	17	771	32,753	0.52	1		
Pauciparous	1,025	20	1,005	42,765	0.47	0.90	0.47–1.72	0.754
Multiparous	570	15	555	23,619	0.64	1.23	0.61–2.45	0.566
ANC visits								
Less than 4 ANC	1,101	27	984	41,933	0.64	1.79	1.01–3.20	0.047
4 ANC and more	1,335	20	1,315	55,801	0.36	1		
Mode of delivery								
Vaginal	2,130	46	2,084	88,688	0.52	1		
Cesarean	253	6	247	10,449	0.57	1.10	0.47–2.59	0.819
Group of maternity								
No free maternity	878	15	863	36,578	0.41	1		
Free maternity	1,505	37	1,468	62,559	0.60	1.44	0.79–2.63	0.232
Overall	2,383	52	2,331	99,137	0.53			

p-d= person-day.

WA = Week of amenorrhea.

HR: hazard ratio.

A low Apgar score at 5 minutes (HR:13.60; p < 0.001), prematurity (HR:2.27; p < 0.001), and LBW (HR:5.89; p < 0.001) were strongly associated as predictors of neonatal mortality (Table 2). Similarly, the mother’s low level of education (None/Primary) (HR:3.26; p < 0.001) and the mother having attended less than 4 ANC visits during the pregnancy (HR:1.79; p < 0.05) were also predictors of neonatal mortality (Table 2).

In bivariate analysis, LBW infants who were born prematurely died more than those born with normal weight (HR: 3.72, 95% CI 1.26 - 10.03, p = 0.003). Infants who were born prematurely and by cesarean section died more than those born vaginally (HR: 2.44, 95% CI 0.59–7.57, p > 0.05), and those were born to mothers who had fewer than four antenatal visits were more likely to die than those mothers had at least four antenatal visits (HR: 2.12, 95% CI 0.72–7.01, p > 0.05) but this difference was not statistically significant.

### Associated factors of death among infants

**Fig 2:**
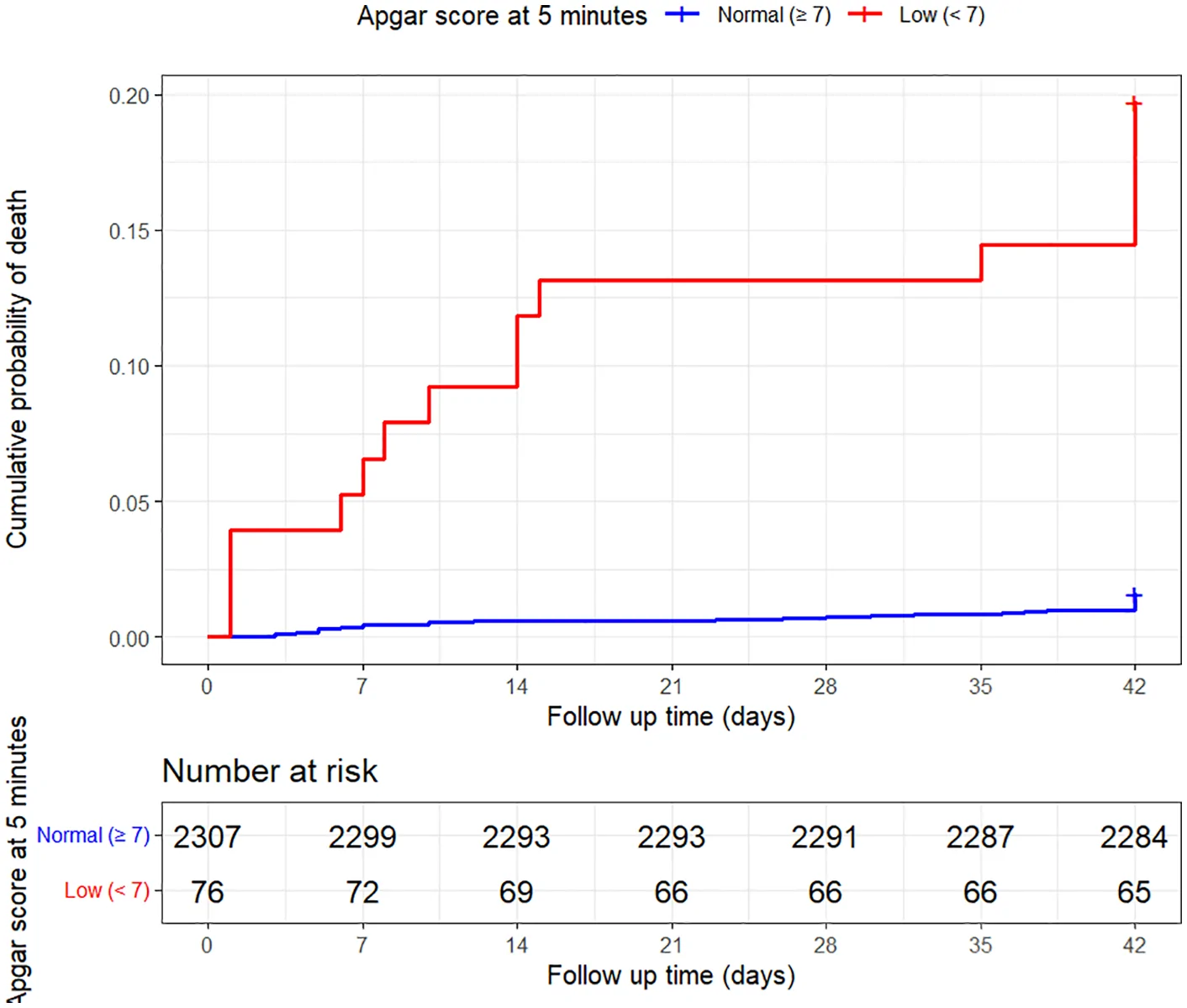
Cumulative incidence of death by Apgar score.

**Fig 3:**
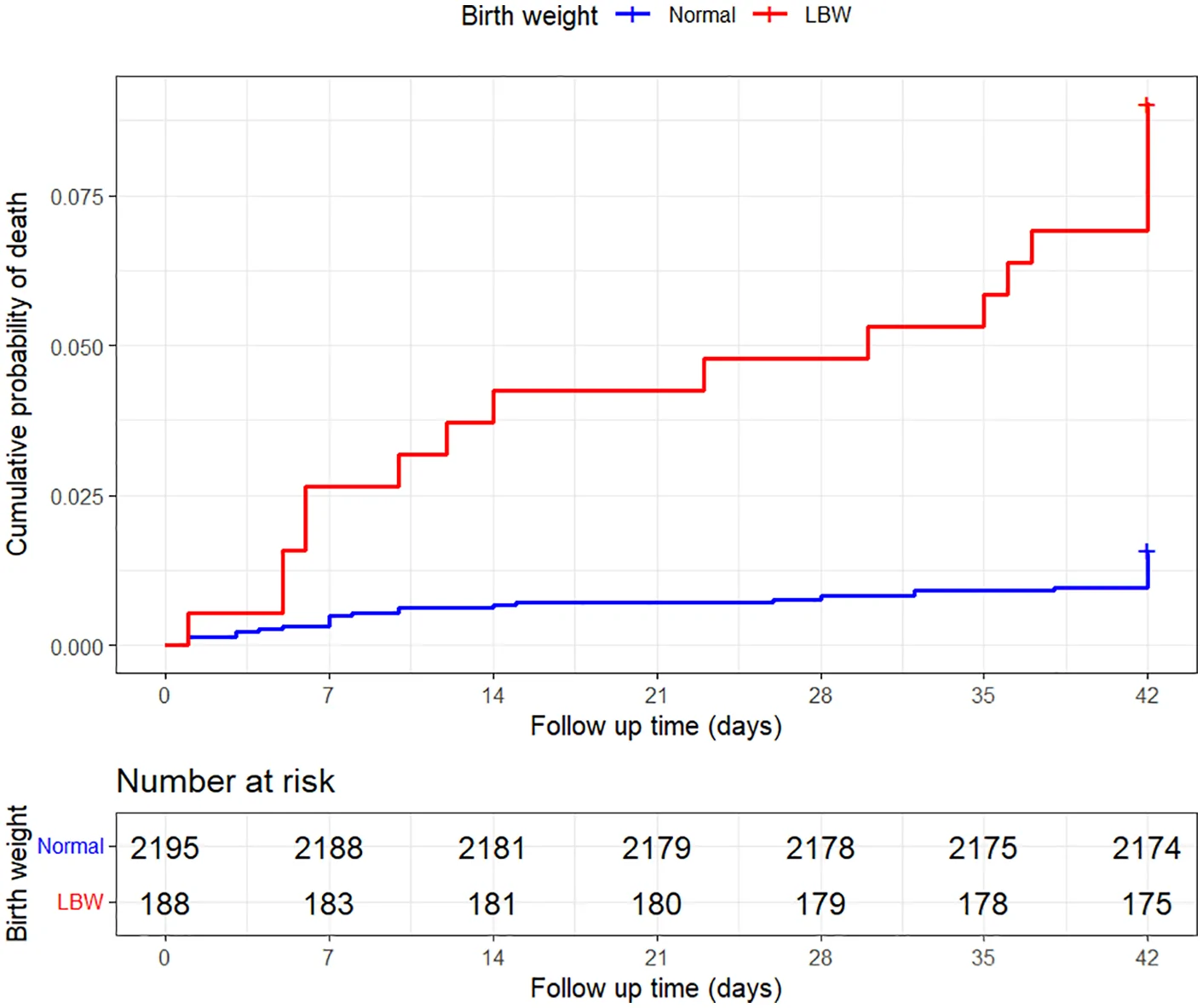
Cumulative incidence of death by birth weight.

**Fig 4:**
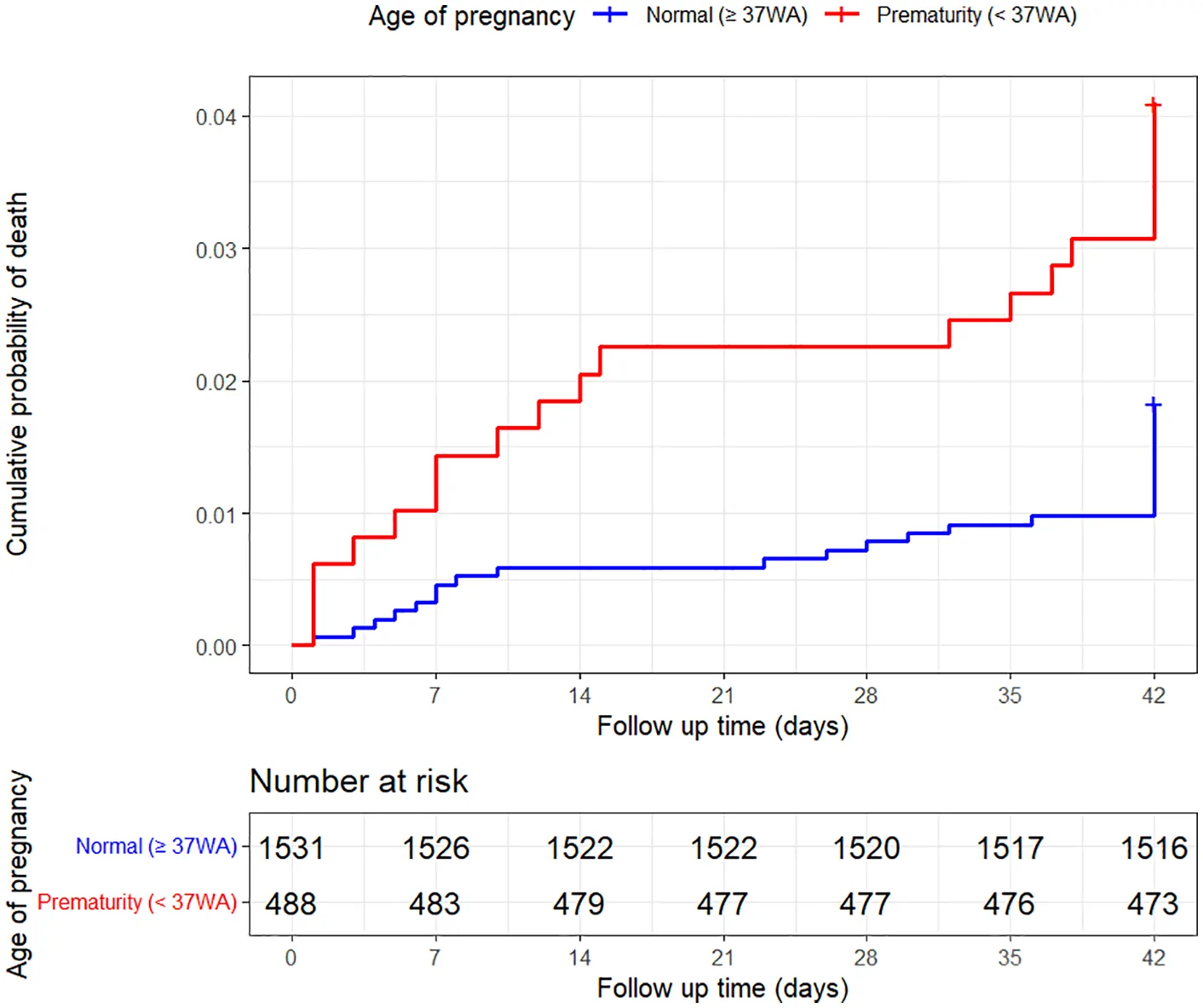
Cumulative incidence of death by age of pregnancy.

After adjustment, the complete case model indicated that infants with a low-5 minutes Apgar scores had a higher risk of death relative to the reference group (Apgar ≥7) (aHR = 9.18; 95% CI: 8.98-9.39), with this association slightly amplified post imputation (aHR = 9.96; 95% CI: 7.01-14.17) (see Fig 2 and Table 3). Similarly, infant with LBW compared to the reference group (birth weight ≥ 2,500g) had a higher risk of death with an aHR of 2.75 (95% CI: 2.14-3.53) in the complete case model and 3.57 (95% CI: 2.39-4.72) in the imputed model (see Fig 3 and Table 3). Prematurity also remained an independent predictor, with a 71% increased risk of death in the complete case model (aHR: 1.71; p < 0.001) and a 97% increased risk in the imputed model (aHR: 1.97; p < 0.01) (see Fig 4 and Table 3).

**Table 3:** Associated factors of early postnatal mortality in Kinshasa from March to July 2024.

Variables	Model with complete case			Model with MICE		
	HRa	p_value	95% CI	HRa	p_value	95% CI
Apgar score at 5 minutes						
< 7	9.18	< 0.001	8.98–9.39	9.96	<0.001	7.01–14.17
≥ 7	Ref			Ref		
Birth weight (gram)						
< 2500	2.75	< 0.001	2.14–3.53	3.57	<0.001	2.39–4.72
>=2500	Ref			Ref		
Gestational age (WA)*						
< 37	1.71	< 0.001	1.38–2.11	1.97	0.003	1.26–3.12
≥ 37	Ref			Ref		
Maternal education						
None/ Primary	2.60	0.126	0.76–8.89	2.46	0.130	0.77–7.90
Secondary/ University	Ref			Ref		
ANC visits*	0.80	< 0.001	0.77–0.82	0.81	0.045	0.66–0.99

* missing data, WA=Week of amenorrhea, aHR: adjusted hazard ratio.

Antenatal care visits were a protective factor in both models with and without imputation. Each additional antenatal consultation was associated with a 19% decrease in the risk of early postnatal mortality (aHR = 0.81, p = 0.045).

## Discussion

In this prospective study, we analyzed the neonatal and maternal factors of early postnatal mortality in a cohort of 2,383 dyads recruited from maternity wards in Kinshasa DRC. In the cohort, 52 children (2.2%) died during the 42 days of follow-up, and the incidence mortality was 0.53 per 1,000 person-days.

The multivariate analysis was conducted using two approaches: an adjusted model based on complete cases and an adjusted model using multiple imputations by the MICE method to handle missing data. The results from both models are generally convergent, with stability in the main major neonatal determinants.

In both the complete case analyses and the multiple imputation analyses, four variables were associated with early postnatal mortality. The multiple imputation analysis identified statistically significant increases in the associations between low-5 minutes Apgar scores, low birthweight, prematurity, and antenatal visits. This difference could be explained by the potential bias related to the exclusion of missing observations in the model without imputation. The imputed model, which takes into account all available information and reduces selection bias, improves the fit and power in the analyzes [28,29]. It is considered more robust and therefore constitutes our main analysis [30,31]. The factors strongly linked to infant mortality were a low Apgar score at the 5th minute, LBW, and prematurity. These factors remained robust in both analyzes, which reinforces the validity of their effects. The fact that mothers attended antenatal consultations, on the other hand, was a survival factor for the infants.

Regarding Apgar score, although the difference is not statistically significant, we found that infants with a low Apgar score were more likely to be registered in secondary/tertiary HCFs than primary ones. This could be explained by the pyramid of the healthcare system in the DRC, where primary facilities provide the minimum package of activities and refer complex cases to higher levels. This referral would explain the concentration of clinically more complex cases in secondary/tertiary health facilities, as observed in other similar contexts [32–34].

In this study, a low-5 minutes Apgar scores was associated with a higher probability of death during the first six weeks of life compared to newborns with a normal score. These results are consistent with the original use of the score created by Dr. Apgar to predict neonatal mortality and support the use of the 5-minute Apgar score in research [16,35,36]. A study conducted in Cameroon by Ndombo et al. and another in Uganda by Nsubuga et al. found that an Apgar score below seven at the 5th minute was a predictor of neonatal and hospital mortality [22,37]. Newborns suffering from severe neonatal asphyxia experience multi-organ failure, including seizures, respiratory distress, and gastrointestinal hemorrhage, which lead to their death. However, this score is often criticized for its subjectivity and lack of reliability in premature newborns, whose clinical signs depend on their physiological maturity [38,39]. Although the Apgar score is subjective and sometimes unreliable, in most LMICs, it can be the only indicator of asphyxia, beside the delay in the initiation of spontaneous breathing [40].

Prematurity was associated with significantly higher mortality compared to full-term newoborns. This could be explained by the fact that prematurity exposes to several complications such as apnea of prematurity due to immaturity of the central respiratory system and respiratory muscles, pulmonary hemorrhage, respiratory distress syndrome due to surfactant deficiency, food intolerance, and hypoglycemia [41]. These conditions increase the risk of neonatal mortality. These results are similar to those reported by Andegiorsh et al. [42] in Eritrea and Mohamed et al. [43] en Ethiopia, who also identified prematurity as a major associated factor in neonatal mortality in intensive care units. Similary, Kebede et Kekulawala in 2021 showed that prematurity increased the risk of early neonatal mortality in tertiary hospitals in Addis Ababa [44]. The health system in DRC remains fragile, marked by recurring health crises (Ebola, Covid-19 et Mpox) and limited availability of infrastructure and qualified personnel. Constraints such as unequal access to care, frequent stockouts of resuscitation equipment, and the limited availability of pediatric intensive care directly impact the survival of newborns, particularly premature infants.

Regarding antenatl visits, each additional antenatal consultation was associated with a 19% decrease in early postnatal mortality. These results are similar to a study conducted in Ethiopia by Kiross et al [45]. Also, a study conducted in 57 LMICs by Doku et al. They found a less than 55% risk of neonatal mortality among women who followed both WHO recommendations (first visit during the 1st trimester and at least four visits during pregnancy) and a less than 32% risk of mortality for those who followed at least one of the WHO recommendations [46]. This was in line with the study conducted in Kenya by Arunda et al., which showed a higher risk of neonatal mortality observed in newborns whose mothers had not attended any antenatal care visits (aOR: 4.0) [47]. This could be explained by the fact that regular visits allow for the early detection and management of maternal complications, while also providing essential preventive interventions such as supplementation and vaccination, thus contributing to reduce risks for the mother and newborn and improving perinatal outcomes. However, this association could be partially influenced by residual factors, such as access to healthcare services or healthcare-seeking behavior, which were not considered in our study..

On the other hand, a study conducted in Burkina Faso by Lansdale et al. in 2024 showed no association between antenatal care (ANC) visits and infant mortality but did show that increased ANC attendance was associated with better growth outcomes at 6 months compared to infants whose mothers had less frequent attendance [48]. Maleya et al. in Lubumbashi, DRC showed that women who had not attended ANC had a high risk of perinatal mortality (OR: 2.70) and LBW (OR: 2.33) [49].

The present study showed that infants born with a LBW were about four times more likely to die compared to newborns with a normal birth weight and premature infants with LBW die more than those born with normal weight (HR: 3.72). This corroborates studies conducted in Ethiopia and Uganda that found high neonatal mortality among children born with low weight compared to those born with normal birth weight [37,50,51]. This mortality can be explained by the fact that infants with LBW have immature organs and systems that expose them to complications such as asphyxia, respiratory distress (respiratory distress syndrome), apnea, and metabolic disorder (such as hypoglycemia). These vulnerabilities increase the risk of infections and malnutrition during childhood, which factors reduce the likelihood of an infant’s survival [52–54].

### Strengths and weaknesses of this study

The limitations of this study lies in the proportion participants lost to follow-up. These losses could introduce selection bias if the missing participants differ systematically from those who completed the follow-up. Another limitation lies in the absence of 5-minute Apgar scores for some newborns, likely due to inadequate documentation rather than a failure to perform the assessment. This gap underscores the need to strengthen the systematic recording of Apgar scores, possibly using digital tools. Furthermore, the dichotomization of certains continuous variables and the absence of others (maternal comorbidities, delivery complications, neonatal resuscitation practices) could introduce residual confounding bias.

Nevertheless, the strengths of this study encompass its prospective cohort design and rigorous statistical methods, thereby making a valuable scientific contribution to the limited data on early postnatal mortality in Africa. Penalized regression methods were used to limit the risk of overfitting due to the low event-to-variable ratio. Furthermore, the multiple imputation approach, used to address missing data and LFTU, reduced biases related to the exclusion of incomplete cases. The benefits of MICE in terms of bias reduction and robustness improvement are based on the assumption that missing data are random. However, some key variables, such as obstetric complications, congenital anomalies, and quality of postnatal care were not available. In addition, although the clustering of facility was taken into account, contextual effects (resources, staffing, protocols) were not modeled. A future study on multilevel analyzes of infants’ mortality according to HCF characteristics by integrating contextual variables would be necessary.

## Conclusion

The predictive factors for infant mortality at 42 days of life were a low Apgar score at 5 minutes, low birth weight, prematurity, and maternal multiparity. ANC attendance was a factor associated with neonatal survival. These results confirm the relevance of the Apgar score as a clinical and prognostic indicator, despite its subjectivity. These findings support prioritizing high-quality ANC coverage, scaling up systematic risk screening during pregnancy and at birth, and ensuring timely identification and clinical management of high-risk newborns in health facilities. They also highlight the need to embed these risk factors into routine clinical decision-making and perinatal surveillance system to guide targeted interventions aimed at reducing neonatal mortality in resource-limited settings. Beyond the policy of free maternity recently implemented in the DRC, broader social protection measures, such as paid maternity leave – as recommended by the International Labour Organization could further contribute to improving neonatal survival by enabling mothers to benefit from adequate postnatal care.

## Supporting information

S1 Table Test of proportional hazards assumption. (DOC)

S2 Table Frequency of missing data by key variables. (DOC)

S1 Appendix Fig A. Test of PH assumption for ANC. Fig B. Test of PH assumption for Apgar at 5 minutes. Fig C. Test of PH assumption for Birthweight. Fig D. Test of PH assumption for gestational age. Fig E. Test of PH assumption for maternal education. Fig F. Goodness of fit of the final model.
(ZIP)
