# Supplementary material for: Impact of maternal and neonatal factors on mortality in the first six weeks of life: A comprehensive cohort study in Kinshasa, DR Congo
Source: PLOS Glob Public Health. 2026 Aug 21;6(8):e0005989. doi: 10.1371/journal.pgph.0005989 (PMC13496423; doi:10.1371/journal.pgph.0005989)
Supplement: Supplementary file 1 [file pgph.0005989.s005.docx]

**Journal Requirements:**

1. In the ethics statement in the Methods, you have specified that verbal consent was obtained. Please provide additional details regarding how this consent was documented and witnessed, and state whether this was approved by the IRB.

Verbal consent was documented by the investigator on the data collection form by checking a specific box confirming that the study information had been provided, that the participant's questions had been satisfactorily answered, and that the participant had voluntarily agreed to participate. No data was collected before obtaining consent. This verbal consent procedure, including its documentation method, was reviewed and approved by the Research Ethics Committee of the School of Public Health at the University of Kinshasa (ESP/CE/152/2023).

We have revised the methods section.

2. Please update your online Competing Interests statement. If you have no competing interests to declare, please state: "The authors have declared that no competing interests exist."

Thank you for this observation, and it has been taken into account.

3. Please note that your Data Availability Statement is currently missing  the DOI/accession number of each dataset. If your manuscript is accepted for publication, you will be asked to provide these details on a very short timeline. We therefore suggest that you provide this information now, though we will not hold up the peer review process if you are unable.

Thank you, and the data availability statement has been noted. Here is the link. <https://doi.org/10.17605/OSF.IO/MYTPA>

4. Please include a separate legend or caption for each main figure in your main manuscript.

Thank you for this relevant observation. This has been taken into account in the new version.

5. If the reviewer comments include a recommendation to cite specific previously published works, please review and evaluate these publications to determine whether they are relevant and should be cited. There is no requirement to cite these works unless the editor has indicated otherwise.

Thank you and well noted

**Reviewer #1**

Thank you for the opportunity to review your manuscript, “Impact of maternal and neonatal factors on mortality in the first six weeks of life: a comprehensive cohort study in Kinshasa City, DR Congo.” The study defines the maternal and newborn factors most associated with newborn mortality within six weeks, concluding that low five-minute Apgar, low birthweight, prematurity, and low maternal education all are associated with worsened newborn mortality risk, while antenatal care attendance was associated with newborn survival. Overall, your substance is important and valuable. I would recommend edits to improve the clarity and directness of your writing, so that readers come away with a clear understanding. Here are specific requests by section:

Front matter:

• It seems overly bold to assert that your study design was a “comprehensive cohort study” when your period of tracking was through six weeks of life (not 10+ years…). I recommend revising to “…: a cohort study in Kinshasa, DR Congo”

Thank you for this relevant observation. This has been taken into account in the new version.

• Is “Kinshasa” different from “Kinshasa City”? If not, go with “Kinshasa” alone.

Kinshasa is no different from Kinshasa city. Thank you, and this has been taken into account in the new version.

• In the Data Availability section, please write out the meaning of OSF.

Thank you. This has been taken into account in the new version.

Abstract
• Why does your list of associated variables not include low maternal education? It seems that this is a clear takeaway from your work, as comes through elsewhere. I’m surprised that your list in the abstract includes multiparity but not low maternal education.

Educational attainment was included in our models due to its documented theoretical importance in the literature. However, in our analyses, whether the model without imputation or the model with imputation, this variable did not show a significant association with the outcome, which is early postnatal mortality. Therefore, it is not included in the list of associated factors presented in the abstract. In contrast, multiparity showed a significant association in the model with imputation (p < 0.05).

In the revised version, after taking into account the reviewers' recommendations to penalize analyses due to the low event-to-variable ratio (EPV = 5.8), the parity variable was no longer included in the new version of the Cox regression.

• The phrase “overall incidence mortality rate…” would better be “overall mortality incidence…” Bigger picture, though, I’m confused about why you list your mortality as “0.53 per 1,000 person-days” rather than “22 deaths per 1,000 live births.” This strikes me as non-intuitive and unlike standard reporting on newborn mortality. Please either explain in the text why you choose to report this way or consider describing your mortality in terms of 1,000 live births.

We expressed neonatal mortality as an incidence rate (0.53 per 1000 person-days) to account for the precise duration of follow-up and exposure time for each infant. This choice of the incidence rate per person-day is based on approaches used in the literature, such as (Bitew et al., 2020). This approach is common in longitudinal analyses. However, to facilitate comparison with standard public health practices, we also report mortality in the classic form, i.e., 22 deaths per 1000 live births. This is taken into account in the new version.

Reference:

Bitew ZW, Alemu A, Ayele EG, Jember DA, Haile MT, Worku T. Incidence Density Rate of Neonatal Mortality and Predictors in Sub-Saharan Africa: A Systematic Review and Meta-Analysis. Int J Pediatr. 2020 Oct 15;2020:3894026. doi: 10.1155/2020/3894026. PMID: 33123204; PMCID: PMC7586147.

• To help your readers to understand what the sentence is about before the parenthetical bits, I recommend reordering your sentence to read “Multivariate Cox regression analysis with multiple imputation identified statistically significant increases in the risk of neonatal mortality for low Apgar score (…)”

Thank you, and this has been taken into account in the new version.

• In general, please look at your sentences and consider whether you could be stating your meaning in a more direct way that will be easily understood by your readers. Most folks will only read the abstract, so you want to be sure that it clearly and directly conveys your main takeaways.

Thank you, and this has been taken into account in the new version.

Introduction

• Try to avoid the informality of contractions like “there’s.” Overall, “there is” and “there are” are considered weak constructions. Rather than “there’s significant geographical disparity…” you could write “…reductions are uneven across geography and age groups, with a 58%...”

Thank you, and this has been taken into account in the new version.

• (Overall) Kindly be careful about your use of “significant.” My preference would be to write “statistically significant” when you mean statistical significance, and to choose a different word when you mean substantial or dramatic – just to avoid reader miscues between statistical and real-world significance.

We have taken care to distinguish in the manuscript the notion of "statistically significant" when referring to the significance in the strict sense of statistical tests, in order to avoid any confusion with a substantial or marked effect in the real world.

• The free maternity policy was specific to Kinshasa, rather than being national across DRC. Kindly revise to clarify this point

Thank you. This has been clarified in the new version. The free public transport policy was not limited to Kinshasa. It was launched in Kinshasa and then extended to other provinces. We have adjusted the text in the new version of the manuscript.

Methods

• Please specify in the text how much data were missing.

Thank you for this observation. The amount of missing data was already specified in the methods section of the manuscript (data analysis subsection).

• Why were 5-minute Apgar scores not available for 47 newborns? Do you believe that this is about Apgar scores not being given, or not being recorded? Is there a practice guideline recommendation to be gleaned from this absence? Would it be appropriate to add this to your limitations?

We found that the 5-minute Apgar score was not available for 47 newborns. This absence appears to be more related to a failure to record the data in the charts or partograms than to a lack of systematic performance of the assessment, which is consistent with the constraints of clinical practice in resource-limited settings.

We have added this text to the new version of the manuscript under the heading of limitations and have formulated a recommendation regarding it.

• Could there have been interactions between any of your variables? Did you test this? I don’t see it, other than a reference to interaction between prenatal visits and gestational age. What about Apgar scores and LBW? Parity and mode of delivery? Etc.

Thank you for this observation. We explored the possibility of interaction between the main variables (e.g., between parity and mode of delivery; Apgar score and low birth weight), but no statistically significant interaction was observed.

We have added the following text to the new version of the manuscript: “The absence of detected interaction suggests that the reported associations are homogeneous across the entire sample, although we cannot rule out the possibility of interactions in other settings or with larger samples.”

• I don’t understand why there would be contamination or why this sentence is necessary: “To minimize the risk of contamination, the 100 HCFs not providing free maternity care were located far from those providing free care.”

Thank you for this observation. We initially used the term "contamination" to indicate the risk that pregnant women attending facilities without a free healthcare policy might go to healthcare facilities that do offer free services. To avoid any confusion, we have removed this line.

• Kindly define that your Apgar scores are five-minute Apgar scores (so that readers will know that you’re talking about five-minute rather than one-minute Apgar scores throughout).

Thank you. This has been integrated into the new version.

• Kindly make your terminology consistent: antenatal care or prenatal care?

Thank you. This has been taken into account in the new version of the manuscript.

Results

• From your work it seems that a main takeaway is that one in five babies with low 5-minute Apgar scores died by the age of six weeks. This is a dramatic finding. Consider stating and calling attention to it more directly.

Thank you for your feedback. This observation has been reformulated and highlighted in the new version to draw attention to it. It has been incorporated into the new version of the manuscript by being included in the abstract and the results section.

• Please use < or > in your p values, rather than equal signs.

Thank you. This has been taken into account in the new version of the manuscript.

• Words in subheadings should be written out, please write out LTFU.

Thank you. This has been taken into account in the new version of the manuscript.

• (Throughout) Are you reporting in the present tense or the past tense? Whichever you choose, please make it consistent.

Thank you for your feedback. We have standardized the verb tenses in the new version of the manuscript.

Discussion

• Given that you’re talking about starting breathing, rather than re-starting something that previously happened, please revise “resumption of spontaneous breathing” to “initiation of spontaneous breathing.”

Thank you. This has been taken into account in the new version of the manuscript.

• Wherever you write “child mortality,” please consider whether it would be more appropriate to write "newborn mortality."

Thank you. This has been taken into account in the new version of the manuscript.

• What is your hypothesis about why antenatal care attendance would be associated with reduced mortality? In addition to situating your work in the literature, it could be helpful to explain a bit about why you think that you’re seeing your findings.

Thank you for your comments. Antenatal care attendance allow for the early screening and management of maternal complications such as gestational diabetes, hypertension, anemia, or infections that can affect the newborn's health. They also offer recommended preventive interventions, such as folic acid and iron supplementation, malaria prophylaxis, and tetanus vaccination, thus contributing to improved perinatal outcomes. Finally, antenatal visits educate pregnant women about warning signs and childbirth preparation, thereby promoting faster access to care in case of complications.

However, this association should not be interpreted as causal, as it may partly reflect underlying differences in socioeconomic status, access to health services, and healthcare-seeking behaviors, which may not have been fully controlled for in our analysis. We have added this nuance to the discussion.

• Please carefully review all of your statistical assertions to verify that they are accurate and appropriate. For example, “The study showed that multiparity of the mother carries a 44% risk of infant’s mortality” will be difficult for readers to digest and could lead to incorrect interpretations.

Thank you, and this has been reformulated in the new version.

• Could multiparity also relate to advanced maternal age, in addition to the accumulation of obstetric risks?

Yes, multiparity could also be linked to advanced maternal age. Advanced maternal age is known to be associated with an increased risk of pregnancy and delivery complications, including gestational hypertension, gestational diabetes, prematurity, and placental abnormalities, which can compromise the newborn's survival.

However, with the newer analyses that take into account the penalized analyses, this parity variable is no longer considered.

References

Voss S, Josefsson A, Ekéus C. Neonatal Outcomes Among Infants of Mothers With Advanced Maternal Age: A National Cohort Study. Acta Paediatr. 2025 Nov;114(11):2893-2899. doi: 10.1111/apa.70185. Epub 2025 Jun 22. PMID: 40545746; PMCID: PMC12520267.

Laopaiboon M, Lumbiganon P, Intarut N, Mori R, Ganchimeg T, Vogel JP, Souza JP, Gülmezoglu AM; WHO Multicountry Survey on Maternal Newborn Health Research Network. Advanced maternal age and pregnancy outcomes: a multicountry assessment. BJOG. 2014 Mar;121(Suppl 1):49-56. doi: 10.1111/1471-0528.12659. PMID: 24641535.

• I recommend “the policy of free maternity care” rather than just “free maternity care” to clarify.

Thank you, and this has been reformulated in the new version.

• I recommend “This could be due to the increase…” rather than “This would be due to the increase…”

Thank you, and this has been reformulated in the new version.

• The sentence “Although multiple imputation was used, some key variables (e.g., obstetric complications, quality of postnatal care) were not available” seems to combine two things. Multiple imputation was how you dealt with the missing data/loss to follow up. Please write a separate sentence about variables that were unavailable.

Thank you, and this has been reformulated in the new version.

• “The follow-up of prenatal visits…” is unclear. Consider instead: “Antenatal care attendance predicted neonatal survival.”

Thank you, and this has been reformulated in the new version.

• I disagree with the second half of the sentence “These results confirm the relevance of the Apgar score as a clinical and prognostic indicator, while also highlighting its limitations due to its subjectivity.” I don’t see evidence of its limitations from your work. Consider instead: “indicator, despite its subjectivity”?

Thank you, and this has been reformulated in the new version.

• I recommend a stronger concluding sentence than “They call for a strengthening of prenatal care, an improvement in the screening and management of at-risk newborns in health facilities, and the integration of these indicators into strategies to reduce neonatal mortality in resource-limited contexts.” The phrases “a strengthening of,” “an improvement in,” and “the integration of” beg for conversion to strong verbs. How, specifically, could you use your findings to reduce neonatal mortality in resource-limited contexts? Say it directly.

Thank you, and this has been reformulated in the new version.

• In the acknowledgements, I suspect that by “exclusively” you mean “especially.”

Thank you, and this has been reformulated in the new version.

**Reviewer #2**

ABSTRACT

Some terminologies are overstated, important methodological details are missing, and some reported effect sizes appear unusually precise relative to the number of events observed. The manuscript alternates between the terms “neonatal mortality,” “infant mortality,” and “mortality at 42 days.” These are not interchangeable. Neonatal mortality conventionally refers to death within the first 28 days of life, infant mortality refers to death before 1 year of age, and the study follows infants for 42 days, so the terminology should therefore be standardized throughout the manuscript. A more accurate phrasing may be: “mortality within the first 42 days of life” or “early postnatal mortality.” This issue is important because inconsistent terminology may mislead readers regarding the scope of the findings.

Thank you for these observations. We have harmonized the terminology throughout the manuscript and opted for "early postnatal mortality".

The adjusted hazard ratios, particularly multiparity (aHR 1.44; 95% CI 1.30–1.59) and Apgar score (aHR 10.26; CI 7.38–14.26), appear unusually precise given that only 52 deaths occurred. With relatively few events, there is concern regarding model overfitting, instability of estimates, and sparse data bias. The authors should report: number of variables included in the final Cox model, events-per-variable ratio, proportional hazards diagnostics, and whether penalized regression methods were considered. This is particularly important because confidence intervals appear narrower than expected for a dataset with only 52 mortality events.

Initially, we included 9 variables in the final model. EPV = 52/9 = 5.8. Due to this low ratio, we completed the analyses using Lasso, and only 5 variables were retained in the final model. This has been added to the methodology section.

Penalized analyses (Lasso/penalized Cox) have been added to the analyses.

The manuscript appears to frame a low Apgar score as an independent causal risk factor. However, the Apgar score is primarily a marker of neonatal compromise rather than a direct cause of mortality, and the language should consistently reflect association rather than causation.

Thank you, and this has been taken into account in the new version. This change has been applied to the summary, results, discussion, and conclusion.

The abstract does not sufficiently clarify follow-up procedures, ascertainment of death (hospital vs home-based confirmation), or loss-to-follow-up, which are critical for interpreting mortality estimates.

Thank you for this feedback. The methods section has been expanded and clarified in the new version.

INTRODUCTION

The sentence: “with a 58% drop in infant and child mortality compared to only 44% for neonatal mortality” is somewhat unclear because “infant and child mortality” combines overlapping age categories, the comparison lacks precision, and the time period is not explicitly repeated. Consider rephrasing for clarity: “Although under-five mortality declined substantially worldwide, reductions in neonatal mortality have progressed more slowly.” The introduction currently moves abruptly from global statistics to regional numbers without explaining why neonatal mortality remains high in the DRC, what healthcare system challenges exist, and why this study is necessary. A stronger contextual paragraph should discuss health system limitations, access to obstetric and neonatal care, socioeconomic barriers, quality of antenatal care, and gaps in follow-up after birth. This would better justify the study rationale. The introduction does not clearly identify a specific knowledge gap. The novelty of a 42-day follow-up cohort in Kinshasa is implied but not explicitly stated, weakening the justification for the study

Thank you for this feedback. The introduction section has been expanded in the new version.

METHODS
The methods section lacks sufficient detail to ensure reproducibility. Recruitment procedures are insufficiently described (e.g., whether consecutive sampling was used, refusal rates, and exclusion criteria). The absence of a participant flow diagram limits transparency regarding selection bias. The sampling strategy across 346 health facilities is unclear. It is not explained how the 95 non-free maternity facilities were selected or whether they were randomly sampled. The statement that they were “as comparable as possible” is subjective and should be replaced with objective criteria. The use of the term “contamination” is inappropriate or unclear in this observational cohort context and requires clarification. Follow-up procedures are insufficiently described. The manuscript does not specify how deaths were ascertained (home visits, verbal autopsy, medical records), nor does it describe handling of loss to follow-up or migration. There is no mention of ethical approval, informed consent procedures, or data confidentiality. Important methodological concern: clustering at the healthcare facility level is not addressed. Given recruitment across 346 facilities, failure to account for intra-cluster correlation may underestimate standard errors and inflate statistical significance. Additionally, definitions of key variables (Apgar scoring standardization, gestational age determination method, and birth weight measurement consistency across facilities) are not sufficiently detailed.

Thank you for this feedback. The methods section has been expanded in the new version.

- For this study, we recorded no refusals but noted 181 losses to follow-up. Newborns with a congenital malformation visible at birth or diagnosed immediately after delivery were not included in the initial cohort.

- The study was approved by the ethics committee of the School of Public Health at the University of Kinshasa (ESP/CE/152/2023).

- Regarding consent, the investigators read a standardized information sheet and recorded the participants' verbal agreement before inclusion. Data confidentiality and the possibility of withdrawing at any time were guaranteed.

- Healthcare facilities were grouped into primary (health centers, referral health centers) and secondary/tertiary (general referral hospitals, university clinics) levels. - Intra-cluster correlation was taken into account in the first version of the manuscript.

- Gestational age was determined by performing at least one ultrasound.

VARIABLES
The outcome definition is appropriate for survival analysis, but it is unclear whether timing of death was exact or interval-censored, and how deaths after discharge were verified. There is insufficient justification for the dichotomization of continuous variables (birth weight, gestational age, Apgar score). This may reduce statistical power and introduce residual confounding. Alternative modeling approaches (e.g., continuous or multi-category variables) should be considered or justified. The inclusion of health facility type (free vs non-free maternity care) is problematic without accounting for clustering at the facility level, and it is unclear whether this is treated as an individual or contextual variable. Important confounders are missing, including maternal comorbidities, delivery complications, socioeconomic status, neonatal resuscitation practices, and congenital anomalies. The time-to-event definition is ambiguous. It is unclear whether time zero is birth or enrollment, which raises concerns about left truncation bias.

This section of the variables has been expanded in the new version of the manuscript.

- The primary outcome was neonatal survival up to six weeks. Time zero corresponds to birth, and the date of death was accurately recorded from hospital records for deaths occurring during the mother's postpartum period in the hospital. For deaths occurring after discharge, the information was verified by telephone follow-up with families through verbal autopsy. Infants who had not died by the end of the follow-up period were censored at the date of the last documented visit or contact.

- As limitations of our study, we added: "The exclusion of certain variables (maternal comorbidities, complications during delivery, neonatal resuscitation practices) could introduce residual confounding bias."

- The dichotomization of continuous variables (Apgar score, birth weight, gestational age) was performed for clinical and readability reasons. However, in the discussion of the limitations, we added that dichotomizing continuous variables could introduce a residual confounding bias.

References for dichotomization:

1. Tavares VB, E Souza JS, Affonso MVG, Da Rocha ES, Rodrigues LFG, da Costa Moraes LF, et al. Factors associated with 5-min APGAR score, death and survival in neonatal intensive care: a case-control study. BMC Pediatr. 2022 Sep 23;22(1):560. doi: 10.1186/s12887-022-03592-9. PMID: 36151512; PMCID: PMC9502588.
2. Yeshaneh A, Kassa A, Kassa ZY, Adane D, Fikadu Y, Wassie ST, et al. The determinants of 5th minute low Apgar score among newborns who delivered at public hospitals in Hawassa City, South Ethiopia. BMC Pediatr. 2021 Jun 8;21(1):266. doi: 10.1186/s12887-021-02745-6. PMID: 34103025; PMCID: PMC8186153.

RESULTS
There is inconsistent separation between unadjusted (bivariate) and adjusted (multivariate) analyses. These should be clearly distinguished. Several hazard ratios appear overly precise relative to the small number of events, particularly in the complete-case model. The extremely narrow confidence interval for Apgar score (e.g., 8.39–9.53) is statistically implausible and suggests potential model instability or reporting error. There is insufficient clarity on whether proportional hazards assumptions were tested. The interpretation of results occasionally uses causal language (“predictors of mortality”) rather than associative language, which is inappropriate for observational data. Subgroup analyses (e.g., cesarean section, prenatal visits) show wide confidence intervals but are interpreted inconsistently.

Thank you for your feedback. This section on variables has been expanded in the new version of the manuscript.

- The proportionality assumptions were tested and found no violations (Table S1).

- Associative language was used instead of causal language.

DISCUSSION
The discussion overstates methodological conclusions regarding multiple imputation. While MICE is appropriate, statements suggesting it “reduces bias” and “improves robustness” should be tempered and conditioned on assumptions of missing at random. The discussion does not sufficiently explain differences between complete-case and imputed models, nor does it identify which variables changed significance and why. The interpretation of the Apgar score is appropriate but sometimes leans toward causal inference. It should consistently be framed as an association. The explanation of referral patterns (higher Apgar scores in secondary/tertiary facilities) is plausible but should be clearly framed as referral bias rather than an epidemiological association. The biological explanation of prematurity is appropriate but overly descriptive; it should be more tightly linked to study findings. There is limited comparison with existing literature beyond citation; effect sizes are not critically compared across studies. The discussion lacks deeper integration into the healthcare context of the Democratic Republic of the Congo, including system-level constraints affecting neonatal survival.

Thank you for your feedback. This section has been expanded in the new version of the manuscript.

CONCLUSION

The conclusion uses causal language (“predictive factors”), which is not appropriate for an observational cohort study. The correct terminology should be “factors associated with mortality.” Multiparity is presented too definitively despite inconsistencies across models and potential sensitivity to imputation. Prenatal care is described as a predictor of survival, but this likely reflects confounding by socioeconomic status and healthcare access and should be softened. The recommendation section is too general and would benefit from more specific, actionable interventions (e.g., neonatal resuscitation training, strengthening referral systems, post-discharge follow-up mechanisms).

Thank you for your feedback. This section has been expanded in the new version of the manuscript.

OVERALL SUMMARY

This is a valuable prospective cohort study addressing an important public health issue in the Democratic Republic of the Congo. The study design and dataset are strengths, particularly the large sample size and follow-up approach. However, major revisions are required due to: inconsistent terminology (neonatal vs infant vs 42-day mortality), insufficient methodological transparency, lack of clustering adjustment, over-precision in statistical estimates, potential overinterpretation of causal relationships, and incomplete reporting of missing data handling and follow-up procedures.

With these issues addressed, the manuscript could make a meaningful contribution to neonatal and maternal health literature in resource-limited settings.

Thank you

**Reviewer #3**

1. Review closely the paper for minor typos/grammar. particularly at lines 39,52,94, 144.

Thank you, and this has been fixed in the new version.

2. The methods section should explicitly elaborate the recruitment of the HCFs and newborns in the study. Let it be clear that the total number of HCFs in the city are considered or just a total of 346. If not the total, then the sampling method of the 346 should be explained. The same for the newborn recruitments. all newborns vs sampled?

Thank you, and this has been fixed in the new version.

3. A little bit of context of the healthcare and characteristics of the city will be helpful.

Thank you, and this has been fixed in the new version.

4. It is important to highlight that the phone call enquires at the HCF about new admission is for the purpose of recruitment into the study.

Thank you, and this has been fixed in the new version.

5. Interesting findings in the discussion section. However, the first 2 paragraphs about the results of the study would be best suited in the results section.
