# Supplementary material for: Impact of maternal and neonatal factors on mortality in the first six weeks of life: A comprehensive cohort study in Kinshasa, DR Congo
Source: PLOS Glob Public Health. 2026 Aug 21;6(8):e0005989. doi: 10.1371/journal.pgph.0005989 (PMC13496423; doi:10.1371/journal.pgph.0005989)
Supplement: Supplementary file 2 [file pgph.0005989.s006.docx]

**Reviewer #1**

Thank you for your revisions. I appreciate your improvements to the paper.

Thank you for enriching it with your suggestions and comments.

- The journal editor should catch some grammatical/typographical inconsistencies, for example the capitalization of “Cox,” punctuation choices, and the phrase “mother and infant dyads…” The word “studies” is not needed at the end of line 106; “were possible” should read “where possible…” on line 158…

Thank you for this relevant observation. This has been taken into account in the new version.

- I wonder whether you would want to add a citation about the free maternity policy for readers who are curious to learn more about it.

This has been taken into account in the new version. We added this to the conclusion: Beyond the policy of free maternity recently implemented in the DRC, broader social protection measures, such as paid maternity leave – as recommended by the International Labour Organization could further contribute to improving neonatal survival by enabling mothers to benefit from adequate postnatal care.

**Reviewer #2**

Thank you. I have reviewed the revised version of the manuscript and carefully assessed the authors' responses to my previous comments. Overall, I am satisfied that my concerns have been adequately addressed, and I believe the revisions have strengthened the manuscript.

Thank you

**Reviewer #3**

The paper is stronger than before, and my previous review comments were addressed.

Thank you
