# Supplementary material for: Impact of maternal and neonatal factors on mortality in the first six weeks of life: A comprehensive cohort study in Kinshasa, DR Congo
Source: PLOS Glob Public Health. 2026 Aug 21;6(8):e0005989. doi: 10.1371/journal.pgph.0005989 (PMC13496423; doi:10.1371/journal.pgph.0005989)
Supplement: Supplementary file 4 [file pgph.0005989.s001.doc]

S1 Table: Test of proportional hazards assumption

|  | rho | chi2 | df | Prob>chi2 |
| --- | --- | --- | --- | --- |
| Apgar score at 5 minutes | -0.24319 | 2.85 | 1 | 0.0912 |
| Birthweight | 0.12142 | 0.73 | 1 | 0.3932 |
| Gestational age (WA) | -0.17256 | 1.37 | 1 | 0.2426 |
| Maternal education | -0.11390 | 0.60 | 1 | 0.4372 |
| ANC visits | 0.19472 | 1.82 | 1 | 0.1779 |
| Global test |  | 5.87 | 5 | 0.3186 |
