# Supplementary material for: Impact of maternal and neonatal factors on mortality in the first six weeks of life: A comprehensive cohort study in Kinshasa, DR Congo
Source: PLOS Glob Public Health. 2026 Aug 21;6(8):e0005989. doi: 10.1371/journal.pgph.0005989 (PMC13496423; doi:10.1371/journal.pgph.0005989)
Supplement: Supplementary file 5 [file pgph.0005989.s002.doc]

S2 Table: Frequency of missing data by key variables

| Variables | n | % |
| --- | --- | --- |
| Birth weight | 0 | 0.00 |
| Gestational age | 364 | 15.27 |
| ANC visits | 37 | 1.65 |
| Maternal education | 0 | 0.00 |
