# Supplementary material for: Impact of maternal and neonatal factors on mortality in the first six weeks of life: A comprehensive cohort study in Kinshasa, DR Congo
Source: PLOS Glob Public Health. 2026 Aug 21;6(8):e0005989. doi: 10.1371/journal.pgph.0005989 (PMC13496423; doi:10.1371/journal.pgph.0005989)
Supplement: Peer review history

# Peer review: Decision Letter 0

**DOI:** 10.1371/journal.pgph.0005989.r001

**Authors:** Abram L. Wagner

8 Jun 2026

PGPH-D-26-00366

Impact of maternal and neonatal factors on mortality in the first six weeks of life: a comprehensive cohort study in Kinshasa City, DR Congo

PLOS Global Public Health

Dear Dr. K. Kabasubabo,

Thank you for submitting your manuscript to PLOS Global Public Health. After careful consideration, we feel that it has merit but does not fully meet PLOS Global Public Health’s publication criteria as it currently stands. Therefore, we invite you to submit a revised version of the manuscript that addresses the points raised during the review process.

Please respond to each reviewer, but you do not necessarily need to provide an edit for each response, as long as you have a clear reason for not doing so.

Please pay attention in particular to using consistent terminology across different parts of your paper, and to limit use of causal language.

Please submit your revised manuscript by Aug 07 2026 11:59PM. If you will need more time than this to complete your revisions, please reply to this message or contact the journal office at globalpubhealth@plos.org. When you're ready to submit your revision, log on to https://www.editorialmanager.com/pgph/ and select the 'Submissions Needing Revision' folder to locate your manuscript file.

Please include the following items when submitting your revised manuscript:

- A letter that responds to each point raised by the editor and reviewer(s). You should upload this letter as a separate file labeled 'Response to Reviewers'.
- A marked-up copy of your manuscript that highlights changes made to the original version. You should upload this as a separate file labeled 'Revised Manuscript with Track Changes'.
- An unmarked version of your revised paper without tracked changes. You should upload this as a separate file labeled 'Manuscript'.

Guidelines for resubmitting your figure files are available below the reviewer comments at the end of this letter.

As the corresponding author, your ORCID iD is verified in the submission system and will appear in the published article. PLOS supports the use of ORCID, and we encourage all coauthors to register for an ORCID iD and use it as well. Please encourage your coauthors to verify their ORCID iD within the submission system before final acceptance, as unverified ORCID iDs will not appear in the published article. *Only*  the individual author can complete the verification step; PLOS staff *cannot*  verify ORCID iDs on behalf of authors.

We look forward to receiving your revised manuscript.

Kind regards,

Abram L. Wagner, PhD, MPH

Academic Editor

PLOS Global Public Health

Journal Requirements:

1. In the ethics statement in the Methods, you have specified that verbal consent was obtained. Please provide additional details regarding how this consent was documented and witnessed, and state whether this was approved by the IRB.

2. Please update your online Competing Interests statement. If you have no competing interests to declare, please state: "The authors have declared that no competing interests exist."

3. Please note that your Data Availability Statement is currently missing  the DOI/accession number of each dataset. If your manuscript is accepted for publication, you will be asked to provide these details on a very short timeline. We therefore suggest that you provide this information now, though we will not hold up the peer review process if you are unable.

4. Please include a separate legend or caption for each main figure in your main manuscript.

5. If the reviewer comments include a recommendation to cite specific previously published works, please review and evaluate these publications to determine whether they are relevant and should be cited. There is no requirement to cite these works unless the editor has indicated otherwise.

Additional Editor Comments (if provided):

[Note: HTML markup is below. Please do not edit.]

Reviewers' comments:

Reviewer's Responses to Questions

**Comments to the Author**

1. Does this manuscript meet PLOS Global Public Health’s publication criteria?

Reviewer #1: Yes

Reviewer #2: Yes

Reviewer #3: Yes

**********

2. Has the statistical analysis been performed appropriately and rigorously?

Reviewer #1: Yes

Reviewer #2: Yes

Reviewer #3: I don't know

**********

3. Have the authors made all data underlying the findings in their manuscript fully available (please refer to the Data Availability Statement at the start of the manuscript PDF file)?

The PLOS Data policy

Reviewer #1: Yes

Reviewer #2: Yes

Reviewer #3: Yes

**********

4. Is the manuscript presented in an intelligible fashion and written in standard English?

Reviewer #1: Yes

Reviewer #2: Yes

Reviewer #3: Yes

**********

Reviewer #1: Thank you for the opportunity to review your manuscript, “Impact of maternal and neonatal factors on mortality in the first six weeks of life: a comprehensive cohort study in Kinshasa City, DR Congo.” The study defines the maternal and newborn factors most associated with newborn mortality within six weeks, concluding that low five-minute Apgar, low birthweight, prematurity, and low maternal education all are associated with worsened newborn mortality risk, while antenatal care attendance was associated with newborn survival. Overall, your substance is important and valuable. I would recommend edits to improve the clarity and directness of your writing, so that readers come away with a clear understanding. Here are specific requests by section:

Front matter:

• It seems overly bold to assert that your study design was a “comprehensive cohort study” when your period of tracking was through six weeks of life (not 10+ years…). I recommend revising to “…: a cohort study in Kinshasa, DR Congo”

• Is “Kinshasa” different from “Kinshasa City”? If not, go with “Kinshasa” alone.

• In the Data Availability section, please write out the meaning of OSF.

Abstract

• Why does your list of associated variables not include low maternal education? It seems that this is a clear takeaway from your work, as comes through elsewhere. I’m surprised that your list in the abstract includes multiparity but not low maternal education.

• The phrase “overall incidence mortality rate…” would better be “overall mortality incidence…” Bigger picture, though, I’m confused about why you list your mortality as “0.53 per 1,000 person-days” rather than “22 deaths per 1,000 live births.” This strikes me as non-intuitive and unlike standard reporting on newborn mortality. Please either explain in the text why you choose to report this way or consider describing your mortality in terms of 1,000 live births.

• To help your readers to understand what the sentence is about before the parenthetical bits, I recommend reordering your sentence to read “Multivariate Cox regression analysis with multiple imputation identified statistically significant increases in the risk of neonatal mortality for low Apgar score (…”

• In general, please look at your sentences and consider whether you could be stating your meaning in a more direct way that will be easily understood by your readers. Most folks will only read the abstract, so you want to be sure that it clearly and directly conveys your main takeaways.

Introduction

• Try to avoid the informality of contractions like “there’s.” Overall, “there is” and “there are” are considered weak constructions. Rather than “there’s significant geographical disparity…” you could write “…reductions are uneven across geography and age groups, with a 58%...”

• (Overall) Kindly be careful about your use of “significant.” My preference would be to write “statistically significant” when you mean statistical significance, and to choose a different word when you mean substantial or dramatic – just to avoid reader miscues between statistical and real-world significance.

• The free maternity policy was specific to Kinshasa, rather than being national across DRC. Kindly revise to clarify this point.

Methods

• Please specify in the text how much data were missing.

• Why were 5-minute Apgar scores not available for 47 newborns? Do you believe that this is about Apgar scores not being given, or not being recorded? Is there a practice guideline recommendation to be gleaned from this absence? Would it be appropriate to add this to your limitations?

• Could there have been interactions between any of your variables? Did you test this? I don’t see it, other than a reference to interaction between prenatal visits and gestational age. What about Apgar scores and LBW? Parity and mode of delivery? Etc.

• I don’t understand why there would be contamination or why this sentence is necessary: “To minimize the risk of contamination, the 100 HCFs not providing free maternity care were located far from those providing free care.”

• Kindly define that your Apgar scores are five-minute Apgar scores (so that readers will know that you’re talking about five-minute rather than one-minute Apgar scores throughout).

• Kindly make your terminology consistent: antenatal care or prenatal care?

Results

• From your work it seems that a main takeaway is that one in five babies with low 5-minute Apgar scores died by the age of six weeks. This is a dramatic finding. Consider stating and calling attention to it more directly.

• Please use < or > in your p values, rather than equal signs.

• Words in subheadings should be written out, please write out LTFU.

• (Throughout) Are you reporting in the present tense or the past tense? Whichever you choose, please make it consistent.

Discussion

• Given that you’re talking about starting breathing, rather than re-starting something that previously happened, please revise “resumption of spontaneous breathing” to “initiation of spontaneous breathing.”

• Wherever you write “child mortality,” please consider whether it would be more appropriate to write "newborn mortality."

• What is your hypothesis about why antenatal care attendance would be associated with reduced mortality? In addition to situating your work in the literature, it could be helpful to explain a bit about why you think that you’re seeing your findings.

• Please carefully review all of your statistical assertions to verify that they are accurate and appropriate. For example, “The study showed that multiparity of the mother carries a 44% risk of infant’s mortality” will be difficult for readers to digest and could lead to incorrect interpretations.

• Could multiparity also relate to advanced maternal age, in addition to the accumulation of obstetric risks?

• I recommend “the policy of free maternity care” rather than just “free maternity care” to clarify.

• I recommend “This could be due to the increase…” rather than “This would be due to the increase…”

• The sentence “Although multiple imputation was used, some key variables (e.g., obstetric complications, quality of postnatal care) were not available” seems to combine two things. Multiple imputation was how you dealt with the missing data/loss to follow up. Please write a separate sentence about variables that were unavailable.

• “The follow-up of prenatal visits…” is unclear. Consider instead: “Antenatal care attendance predicted neonatal survival.”

• I disagree with the second half of the sentence “These results confirm the relevance of the Apgar score as a clinical and prognostic indicator, while also highlighting its limitations due to its subjectivity.” I don’t see evidence of its limitations from your work. Consider instead: “indicator, despite its subjectivity” ?

• I recommend a stronger concluding sentence than “They call for a strengthening of prenatal care, an improvement in the screening and management of at-risk newborns in health facilities, and the integration of these indicators into strategies to reduce neonatal mortality in resource-limited contexts.” The phrases “a strengthening of,” “an improvement in,” and “the integration of” beg for conversion to strong verbs. How, specifically, could you use your findings to reduce neonatal mortality in resource-limited contexts? Say it directly.

• In the acknowledgements, I suspect that by “exclusively” you mean “especially.”

Reviewer #2: Dear Author,

Please find the feedback on your manuscript below. Thank you for the opportunity to review your work.

ABSTRACT

Some terminologies are overstated, important methodological details are missing, and some reported effect sizes appear unusually precise relative to the number of events observed. The manuscript alternates between the terms “neonatal mortality,” “infant mortality,” and “mortality at 42 days.” These are not interchangeable. Neonatal mortality conventionally refers to death within the first 28 days of life, infant mortality refers to death before 1 year of age, and the study follows infants for 42 days, so the terminology should therefore be standardized throughout the manuscript. A more accurate phrasing may be: “mortality within the first 42 days of life” or “early postnatal mortality.” This issue is important because inconsistent terminology may mislead readers regarding the scope of the findings. The adjusted hazard ratios, particularly multiparity (aHR 1.44; 95% CI 1.30–1.59) and Apgar score (aHR 10.26; CI 7.38–14.26), appear unusually precise given that only 52 deaths occurred. With relatively few events, there is concern regarding model overfitting, instability of estimates, and sparse data bias. The authors should report: number of variables included in the final Cox model, events-per-variable ratio, proportional hazards diagnostics, and whether penalized regression methods were considered. This is particularly important because confidence intervals appear narrower than expected for a dataset with only 52 mortality events. The manuscript appears to frame a low Apgar score as an independent causal risk factor. However, the Apgar score is primarily a marker of neonatal compromise rather than a direct cause of mortality, and the language should consistently reflect association rather than causation. The abstract does not sufficiently clarify follow-up procedures, ascertainment of death (hospital vs home-based confirmation), or loss-to-follow-up, which are critical for interpreting mortality estimates.

INTRODUCTION

The sentence: “with a 58% drop in infant and child mortality compared to only 44% for neonatal mortality” is somewhat unclear because “infant and child mortality” combines overlapping age categories, the comparison lacks precision, and the time period is not explicitly repeated. Consider rephrasing for clarity: “Although under-five mortality declined substantially worldwide, reductions in neonatal mortality have progressed more slowly.” The introduction currently moves abruptly from global statistics to regional numbers without explaining why neonatal mortality remains high in the DRC, what healthcare system challenges exist, and why this study is necessary. A stronger contextual paragraph should discuss health system limitations, access to obstetric and neonatal care, socioeconomic barriers, quality of antenatal care, and gaps in follow-up after birth. This would better justify the study rationale. The introduction does not clearly identify a specific knowledge gap. The novelty of a 42-day follow-up cohort in Kinshasa is implied but not explicitly stated, weakening the justification for the study.

METHODS

The methods section lacks sufficient detail to ensure reproducibility. Recruitment procedures are insufficiently described (e.g., whether consecutive sampling was used, refusal rates, and exclusion criteria). The absence of a participant flow diagram limits transparency regarding selection bias. The sampling strategy across 346 health facilities is unclear. It is not explained how the 95 non-free maternity facilities were selected or whether they were randomly sampled. The statement that they were “as comparable as possible” is subjective and should be replaced with objective criteria. The use of the term “contamination” is inappropriate or unclear in this observational cohort context and requires clarification. Follow-up procedures are insufficiently described. The manuscript does not specify how deaths were ascertained (home visits, verbal autopsy, medical records), nor does it describe handling of loss to follow-up or migration. There is no mention of ethical approval, informed consent procedures, or data confidentiality. Important methodological concern: clustering at the healthcare facility level is not addressed. Given recruitment across 346 facilities, failure to account for intra-cluster correlation may underestimate standard errors and inflate statistical significance. Additionally, definitions of key variables (Apgar scoring standardization, gestational age determination method, and birth weight measurement consistency across facilities) are not sufficiently detailed.

VARIABLES

The outcome definition is appropriate for survival analysis, but it is unclear whether timing of death was exact or interval-censored, and how deaths after discharge were verified. There is insufficient justification for the dichotomization of continuous variables (birth weight, gestational age, Apgar score). This may reduce statistical power and introduce residual confounding. Alternative modeling approaches (e.g., continuous or multi-category variables) should be considered or justified. The inclusion of health facility type (free vs non-free maternity care) is problematic without accounting for clustering at the facility level, and it is unclear whether this is treated as an individual or contextual variable. Important confounders are missing, including maternal comorbidities, delivery complications, socioeconomic status, neonatal resuscitation practices, and congenital anomalies. The time-to-event definition is ambiguous. It is unclear whether time zero is birth or enrollment, which raises concerns about left truncation bias.

RESULTS

There is inconsistent separation between unadjusted (bivariate) and adjusted (multivariate) analyses. These should be clearly distinguished. Several hazard ratios appear overly precise relative to the small number of events, particularly in the complete-case model. The extremely narrow confidence interval for Apgar score (e.g., 8.39–9.53) is statistically implausible and suggests potential model instability or reporting error. There is insufficient clarity on whether proportional hazards assumptions were tested. The interpretation of results occasionally uses causal language (“predictors of mortality”) rather than associative language, which is inappropriate for observational data. Subgroup analyses (e.g., cesarean section, prenatal visits) show wide confidence intervals but are interpreted inconsistently.

DISCUSSION

The discussion overstates methodological conclusions regarding multiple imputation. While MICE is appropriate, statements suggesting it “reduces bias” and “improves robustness” should be tempered and conditioned on assumptions of missing at random. The discussion does not sufficiently explain differences between complete-case and imputed models, nor does it identify which variables changed significance and why. The interpretation of the Apgar score is appropriate but sometimes leans toward causal inference. It should consistently be framed as an association. The explanation of referral patterns (higher Apgar scores in secondary/tertiary facilities) is plausible but should be clearly framed as referral bias rather than an epidemiological association. The biological explanation of prematurity is appropriate but overly descriptive; it should be more tightly linked to study findings. There is limited comparison with existing literature beyond citation; effect sizes are not critically compared across studies. The discussion lacks deeper integration into the healthcare context of the Democratic Republic of the Congo, including system-level constraints affecting neonatal survival.

CONCLUSION

The conclusion uses causal language (“predictive factors”), which is not appropriate for an observational cohort study. The correct terminology should be “factors associated with mortality.” Multiparity is presented too definitively despite inconsistencies across models and potential sensitivity to imputation. Prenatal care is described as a predictor of survival, but this likely reflects confounding by socioeconomic status and healthcare access and should be softened. The recommendation section is too general and would benefit from more specific, actionable interventions (e.g., neonatal resuscitation training, strengthening referral systems, post-discharge follow-up mechanisms).

OVERALL SUMMARY

This is a valuable prospective cohort study addressing an important public health issue in the Democratic Republic of the Congo. The study design and dataset are strengths, particularly the large sample size and follow-up approach. However, major revisions are required due to: inconsistent terminology (neonatal vs infant vs 42-day mortality), insufficient methodological transparency, lack of clustering adjustment, over-precision in statistical estimates, potential overinterpretation of causal relationships, and incomplete reporting of missing data handling and follow-up procedures.

With these issues addressed, the manuscript could make a meaningful contribution to neonatal and maternal health literature in resource-limited settings.

Thank you

Reviewer #3: Thanks to the authors for a great work put into this manuscript. I have a few comment that can hopefully improve the strength of the paper.

1. Review closely the paper for minor typos/grammar. particularly at lines 39,52,94, 144.

2. The methods section should explicitly elaborate the recruitment of the HCFs and newborns in the study. Let it be clear that the total number of HCFs in the city are considered or just a total of 346. If not the total, then the sampling method of the 346 should be explained. The same for the newborn recruitments. all newborns vs sampled?

3. A little bit of context of the healthcare and characteristics of the city will be helpful.

4. It is important to highlight that the phone call enquires at the HCF about new admission is for the purpose of recruitment into the study.

5. Interesting findings in the discussion section. However, the first 2 paragraphs about the results of the study would be best suited in the results section.

**********

what does this mean?). If published, this will include your full peer review and any attached files.

**Do you want your identity to be public for this peer review?**  If you choose “no”, your identity will remain anonymous but your review may still be made public.

For information about this choice, including consent withdrawal, please see our Privacy Policy

Reviewer #1: No

Reviewer #2: No

Reviewer #3: No

**********

[NOTE: If reviewer comments were submitted as an attachment file, they will be attached to this email and accessible via the submission site. Please log into your account, locate the manuscript record, and check for the action link "View Attachments". If this link does not appear, there are no attachment files.]

# Author comment: Author response to Decision Letter 1

**DOI:** 10.1371/journal.pgph.0005989.r002

1 Jul 2026

Attachment Submitted filename: Response to reviewers.docx

# Peer review: Decision Letter 1

**DOI:** 10.1371/journal.pgph.0005989.r003

**Authors:** Emma Campbell

29 Jul 2026

PGPH-D-26-00366R1

Impact of maternal and neonatal factors on mortality in the first six weeks of life: a cohort study in Kinshasa, DR Congo

PLOS Global Public Health

Dear Dr. K. Kabasubabo,

Thank you for submitting your manuscript to PLOS Global Public Health. After careful consideration, we feel that it has merit but does not fully meet PLOS Global Public Health’s publication criteria as it currently stands. Therefore, we invite you to submit a revised version of the manuscript that addresses the points raised during the review process.

The reviewers have assessed the revised manuscript and have requested some minor final revisions before this can proceed. Please review their comments below and make the appropriate revisions before you resubmit.

Please submit your revised manuscript by Aug 27 2026 11:59PM. If you will need more time than this to complete your revisions, please reply to this message or contact the journal office at globalpubhealth@plos.org. When you're ready to submit your revision, log on to https://www.editorialmanager.com/pgph/ and select the 'Submissions Needing Revision' folder to locate your manuscript file.

Please include the following items when submitting your revised manuscript:

- A letter that responds to each point raised by the editor and reviewer(s). You should upload this letter as a separate file labeled 'Response to Reviewers'.
- A marked-up copy of your manuscript that highlights changes made to the original version. You should upload this as a separate file labeled 'Revised Manuscript with Track Changes'.
- An unmarked version of your revised paper without tracked changes. You should upload this as a separate file labeled 'Manuscript'.

Guidelines for resubmitting your figure files are available below the reviewer comments at the end of this letter.

As the corresponding author, your ORCID iD is verified in the submission system and will appear in the published article. PLOS supports the use of ORCID, and we encourage all coauthors to register for an ORCID iD and use it as well. Please encourage your coauthors to verify their ORCID iD within the submission system before final acceptance, as unverified ORCID iDs will not appear in the published article. *Only*  the individual author can complete the verification step; PLOS staff *cannot*  verify ORCID iDs on behalf of authors.

We look forward to receiving your revised manuscript.

Kind regards,

Emma Campbell, Ph.D

Staff Editor

PLOS Global Public Health

**Journal Requirements:**

If the reviewer comments include a recommendation to cite specific previously published works, please review and evaluate these publications to determine whether they are relevant and should be cited. There is no requirement to cite these works unless the editor has indicated otherwise.

Please review your reference list to ensure that it is complete and correct. If you have cited papers that have been retracted, please include the rationale for doing so in the manuscript text, or remove these references and replace them with relevant current references. Any changes to the reference list should be mentioned in the rebuttal letter that accompanies your revised manuscript. If you need to cite a retracted article, indicate the article’s retracted status in the References list and also include a citation and full reference for the retraction notice.

**Additional Editor Comments (if provided):**

[Note: HTML markup is below. Please do not edit.]

Reviewers' comments:

Reviewer's Responses to Questions

**Comments to the Author**

Reviewer #1: All comments have been addressed

Reviewer #2: All comments have been addressed

Reviewer #3: All comments have been addressed

**********

publication criteria?

Reviewer #1: Yes

Reviewer #2: Yes

Reviewer #3: Yes

**********

3. Has the statistical analysis been performed appropriately and rigorously?

Reviewer #1: Yes

Reviewer #2: Yes

Reviewer #3: Yes

**********

4. Have the authors made all data underlying the findings in their manuscript fully available (please refer to the Data Availability Statement at the start of the manuscript PDF file)?

The PLOS Data policy

Reviewer #1: Yes

Reviewer #2: Yes

Reviewer #3: Yes

**********

5. Is the manuscript presented in an intelligible fashion and written in standard English?

Reviewer #1: Yes

Reviewer #2: Yes

Reviewer #3: Yes

**********

**Reviewer #1:**  Thank you for your revisions. I appreciate your improvements to the paper.

My final comments are very minor:

- The journal editor should catch some grammatical/typographical inconsistencies, for example the capitalization of “Cox,” punctuation choices, and the phrase “mother and infant dyads…” The word “studies” is not needed at the end of line 106; “were possible” should read “where possible…” on line 158…

- I wonder whether you would want to add a citation about the free maternity policy for readers who are curious to learn more about it.

**Reviewer #2:**  Thank you. I have reviewed the revised version of the manuscript and carefully assessed the authors' responses to my previous comments. Overall, I am satisfied that my concerns have been adequately addressed, and I believe the revisions have strengthened the manuscript.

**Reviewer #3** : The paper is stronger than before, and my previous review comments were addressed.

**********

what does this mean?). If published, this will include your full peer review and any attached files.

**Do you want your identity to be public for this peer review?**  If you choose “no”, your identity will remain anonymous but your review may still be made public.

For information about this choice, including consent withdrawal, please see our Privacy Policy

Reviewer #1: No

Reviewer #2: No

Reviewer #3: No

**********

[NOTE: If reviewer comments were submitted as an attachment file, they will be attached to this email and accessible via the submission site. Please log into your account, locate the manuscript record, and check for the action link "View Attachments". If this link does not appear, there are no attachment files.]

**Figure Resubmissions:**

# Author comment: Author response to Decision Letter 2

**DOI:** 10.1371/journal.pgph.0005989.r004

30 Jul 2026

Attachment Submitted filename: Response_to_reviewers_auresp_2.docx

# Editor report: Decision Letter 2

**DOI:** 10.1371/journal.pgph.0005989.r005

**Authors:** Julia Robinson

4 Aug 2026

Impact of maternal and neonatal factors on mortality in the first six weeks of life: a cohort study in Kinshasa, DR Congo

PGPH-D-26-00366R2

Dear Docteur K. Kabasubabo,

We are pleased to inform you that your manuscript 'Impact of maternal and neonatal factors on mortality in the first six weeks of life: a cohort study in Kinshasa, DR Congo' has been provisionally accepted for publication in PLOS Global Public Health.

Before your manuscript can be formally accepted you will need to complete some formatting changes, which you will receive in a follow up email. A member of our team will be in touch with a set of requests.

Please note that your manuscript will not be scheduled for publication until you have made the required changes, so a swift response is appreciated.

IMPORTANT: The editorial review process is now complete. PLOS will only permit corrections to spelling, formatting or significant scientific errors from this point onwards. Requests for major changes, or any which affect the scientific understanding of your work, will cause delays to the publication date of your manuscript.

If your institution or institutions have a press office, please notify them about your upcoming paper to help maximize its impact. If they'll be preparing press materials, please inform our press team as soon as possible -- no later than 48 hours after receiving the formal acceptance. Your manuscript will remain under strict press embargo until 2 pm Eastern Time on the date of publication. For more information, please contact globalpubhealth@plos.org.

Thank you again for supporting Open Access publishing; we are looking forward to publishing your work in PLOS Global Public Health.

Best regards,

Julia Robinson

Executive Editor

PLOS Global Public Health

***********************************************************

Reviewer Comments (if any, and for reference):
